# Young Swimmers’ Middle-Distance Performance Variation within a Training Season

**DOI:** 10.3390/ijerph18031010

**Published:** 2021-01-24

**Authors:** Sara Ferreira, Diogo Duarte Carvalho, Ricardo Cardoso, Manoel Rios, Susana Soares, Argyris Toubekis, Ricardo J. Fernandes

**Affiliations:** 1 Centre of Research, Education, Innovation and Intervention in Sport, Faculty of Sport, University of Porto, 4200-450 Porto, Portugal; sara_ferreira_1120@hotmail.com (S.F.); diogoduarte_03@hotmail.com (D.D.C.); ricardocardoso.coach@gmail.com (R.C.); manoel.rios@hotmail.com (M.R.); susana@fade.up.pt (S.S.); 2 Porto Biomechanics Laboratory, University of Porto, 4200-450 Porto, Portugal; 3 School of Physical Education & Sport Science, National and Kapodistrian University of Athens, 17233 Athens, Greece; atoubekis@phed.uoa.gr

**Keywords:** macrocycle, swimming, lactate, glucose, technique

## Abstract

The current study aimed to longitudinally evaluate anthropometric, physiological, and biomechanical variables related to middle-distance performance during a 45-week swimming training season. Thirty-four swimmers (age: 12.07 ± 1.14 years) performed a maximum of 400 m front crawl at the beginning (T1) and finish of the first macrocycle (T2, 15 weeks) and the finish of the second (T3, 18 weeks) and third macrocycles (T4, 12 weeks). Time-related variables, stroke rate (SR), stroke length (SL), and stroke index (SI) were recorded during the test, and blood lactate ([La]) and glucose ([Glu]) concentrations were measured post-exercise. The time of the 400 m effort decreased after each macrocycle (T2 vs. T1, 7.8 ± 5.6%; T3 vs. T2, 3.7 ± 3.1%; T4 vs. T3, 3.8 ± 3.4%; *p* < 0.01). Four hundred meter speed changes between T1 and T2 were positively related to variations in [La], [Glu], SL, and SI (*r* = 0.36–0.60, *p* < 0.05). Changes between T2 and T3 were related to SI only (*r* = 0.5, *p* < 0.05), and modifications between T3 and T4 were associated with SL and SI variations (*r* = 0.34 and 0.65, *p* < 0.05). These results indicate that a well-structured year plan including three macrocycles leads to a significant age-group swimming performance improvement, mostly connected with an increase in technical proficiency.

## 1. Introduction

Swimmers are involved in training and competition at very young ages [1,2], and appropriate training planning, as well as regular testing, should be applied within a training season [3,4]. Such an approach aims to guide the development of energetic and biomechanical attributes, leading to improved physical conditioning, skill acquisition, and performance enhancement [5,6]. Short, medium, or long training planning should consider the characteristics and specific needs of young swimmers and apply proper periodization. As such, a year plan is divided into shorter periods, normally using one of three periodization designs, including one, two, or three macrocycles. Regarding the three-macrocycle periodization model (each ending with a main competition), the transition period is aligned with school holidays [7]. Due to the connection with the school activities calendar, this specific periodization is likely one of the best suited for young swimmers [8].

To achieve the optimal performance in main competitions, the training load needs to be gradual and specific. It is expected that swimmers improve their performance after each training season by 1% in the elite level [9] and by up to 10% in younger swimmers due to growth and performance level [10]. Specialization in young swimmers may occur between 11 and 13 years and 13 and 15 years for girls and boys, respectively [11]. Unfortunately, due to scarce research on the topic, there are limited specific directions on training periodization of children and young swimmers. Moreover, during childhood and youth, the progression of maturation affects performance and influences not only physiological variables (e.g., cardiovascular adaptations [8,12]) but also biomechanical variables (e.g., stroke rate, stroke length, and stroke index (SR, SL, and SI, respectively)) [13,14]. Although cross-sectional studies have focused on the importance of anthropometric, physiological, and biomechanical variables in young swimmers’ performance [14,15,16], few longitudinal studies are directed toward evaluating physiological and biomechanical changes in young swimmers. Even so, some studies have applied interventions over a macrocycle [3,10,17] or a training season [8,15], relating anthropometric, physiological, and biomechanical changes to swimming performance.

For facilitating training periodization, planning, and evaluation of physiological and biomechanical changes over a macrocycle [9] (or repeated macrocycles), valid testing is a prerequisite. In this context, the 400 m maximum effort has been suggested for testing young swimmers, getting attention in swimming research [4,8,10] since it is regularly used to evaluate the aerobic power of swimmers [10,18,19]. Moreover, training control is a primordial task of the coach when the aim is to improve performance in the main competitions [20,21]. Appropriate testing, particularly focusing on physiological (e.g., blood lactate concentrations and oxygen uptake) and biomechanical (kinetics and kinematics of the upper and lower limbs’ actions) evaluations, helps understand the effect of the training volume and intensity on competitive performance [3,16,22,23,24]. The purpose of the current study was to describe the evolution of middle-distance swimming performance along with physiological and biomechanical changes in young swimmers during a training season including three macrocycles. We hypothesized that changes in physiological and biomechanical variables are directly related to improved performance during the training season.

## 2. Materials and Methods

Thirty-four competitive swimmers (10 girls and 24 boys) aged between 9 and 14 years, with ≥ four land and in-water training sessions per week, participated in the current study. Swimmers with ≤ one year of competitive experience were not included. The main characteristics of the participants, including chronological age and maturation stage (verified by a valid and reliable self-assessment of secondary sexual characteristics; [25]), are presented in Table 1.

The swimmers were tested at four moments of the training season: at the beginning (T1) and finish (T2) of the first macrocycle and at the finish of the second (T3) and third (T4) macrocycles. The duration of the first, second, and third macrocycles was 15, 18, and 12 weeks, respectively. The closing of each macrocycle coincided with the important competitions of the season. The training content of each macrocycle is demonstrated in Table 2. At each testing moment, the swimmers were asked to perform a maximum of 400 m front crawl effort, with the performance time in each 100 m split and total 400 m time being recorded by qualified timekeepers (Seiko, Tokyo, Japan).

The heart rate (HR) was recorded using telemetry (Polar Electro, Kempele, Finland) during the recovery period immediately after the 400 m front crawl effort (at 10, 30, 60, and 120 s). Lactate ([La]; Lactate Pro, Arkay, Inc., Kyoto, Japan) and glucose ([Glu]) concentrations (GlucocardTM, A. Menarini, Paço de Arcos, Portugal) were measured during the third minute of recovery using two capillary blood samples from the swimmer’s finger. The rating of perceived exertion (RPE) was recorded at the end of the 400 m effort on a 20-point scale. To analyze biomechanical variables, a video recorder (HDR-CX160E 60 Hz, Sony, Tokyo, Japan) was placed strategically (above the water and perpendicular to the swimmer direction at a 10 m distance from the swimming pool). The SR was calculated in the last 25 m of each 100 m split by the time taken to complete three consecutive upper-limb cycles (Kinovea software 8.15, Bordeaux, France). The SR assessment was repeated two times (and used the mean value) for assuring reliability. The stroke length (SL) was assessed by the quotient of mean speed with the mean SR [17], and the SI was calculated as the product of the mean SL and the 400 m mean speed [17]. The diet was controlled by asking swimmers’ parents to maintain a similar nutritional content the day before each testing session.

### Statistical Analysis

Normal distribution was tested using the Kolmogorov–Smirnov test, and sphericity was verified using the Mauchly test. When the assumption of sphericity was not met, the significance of *F*-ratios was adjusted according to the Greenhouse–Geisser procedures. To compare the physiological and biomechanical variables, we used a repeated-measures one-way analysis of variance (ANOVA). Furthermore, to compare HR recovery (four testing moments × four points of recovery) and changes in swimming time, SR, SL, and SI in each 100 m lap of the 400 m test, we used the two-way repeated-measures ANOVA. Analysis of covariance was applied using the maturation stage and body mass index as covariates. A Tukey honest significant difference post-hoc test was used to compare means when significant *F*-ratios were found. Cohen’s effect size (*d*) was calculated as the mean differences divided by the pooled standard deviation and characterized as small (<0.20), medium (0.2–0.8), and large (>0.8) [26]. The 95% confidence intervals (95% CI) were also calculated. To analyze relations between variables, we used the Pearson correlation coefficient. The data were shown as mean ± standard deviation, and statistical significance was set at *p* < 0.05.

## 3. Results

### 3.1. Changes in 400 m Time

**Figure 1:**
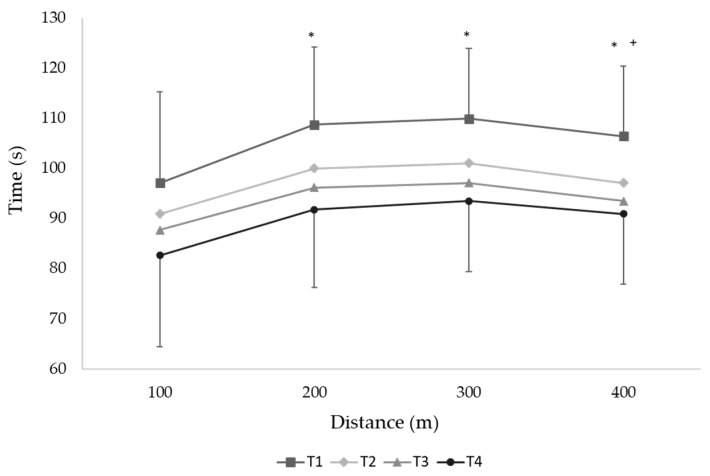
Time for each 100 m split in the 400 m front crawl effort at testing moments T1 (beginning of the first macrocycle) and T2, T3, and T4 (end of the first, second, and third macrocycles, respectively). * and +: *p* < 0.05 compared to 100 m partial and to 200 and 300 m partials.

Since no interaction of sex and performance time changes observed between macrocycles (F_3.96_ = 0.105, *p* = 0.96), the performance variation was tested with male and female swimmers grouped in the same sample. The analysis of covariance indicated that the maturation stage was a relevant predictor of the 400 m performance time changes (*F*_1.31_ = 4.67, *p* = 0.04) and body mass index changes did not interfere with the 400 m time at each testing moment (*F*_1.31_ = 0.34, *p* = 0.56) or in performance changes between testing moments (*p* > 0.05). Young swimmers improved their performance across the year, with the 400 m front crawl time decreasing from T4 compared to T1 (mean ± SD, [95% CI]; 14.6 ± 5.9%; 12.5, 16.7%; *F*_3.99_ = 89.8, *p* < 0.001, Table 3). Moreover, the 400 m time decreased after each macrocycle (T2 vs. T1, 7.8 ± 5.6%, 5.8, 9.7%; T3 vs. T2, 3.7 ± 3.1%; 2.6, 4.8%, and T4 vs. T3, 3.8 ± 3.4%; 2.6, 5.0%; *p* < 0.01, Table 3). The time in each 100 m split in the 400 m effort decreased in each successive test from T1 to T4 (Figure 1).

### 3.2. Changes in Physiological Variables and Rating of Perceived Exertion Following 400 m

There was no difference between testing moments in the post-400 m HR (*F*_3.99_ = 1.07; *p* = 0.36, Table 3). Moreover, the HR was decreasing over the recovery period following each 400 m test (from 10–120 s) without any difference in the rate of recovery between testing moments. Following T3 and T4 testing moments, the post-400 m [La] increased compared to T1 (*p* < 0.05). An increment was also observed after T4 compared to T2 (*F*_3.99_ = 8.5; *p* < 0.01; Table 3). Blood [Glu] increased in T3 compared to T2 and T1 and in T4 compared to T2 (*F*_3.99_ = 11.43; *p* < 0.01, Table 3). There was no difference in the ratings of perceived exertion between the four testing moments (*F*_3.99_ = 0.49; *p* = 0.68, Table 3).

### 3.3. Changes in Technique and Anthropometry

**Figure 2:**
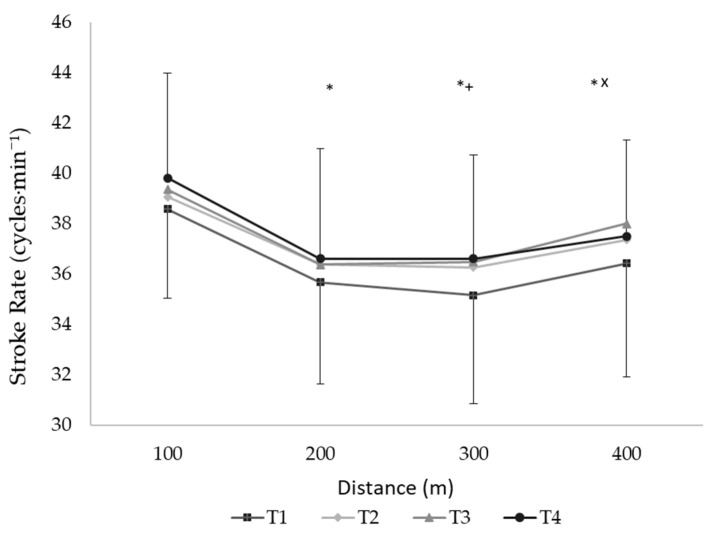
The stroke rate in each 100 m split of the 400 m front crawl effort at testing moments T1, T2, T3, and T4. T1: beginning of the first macrocycle; T2, T3, and T4: end of the first, second, and third macrocycles, respectively. * and +: *p* < 0.05 compared to 100 m and 400 m. ^x^: *p* < 0.05 compared to 200 m partial.

**Figure 3:**
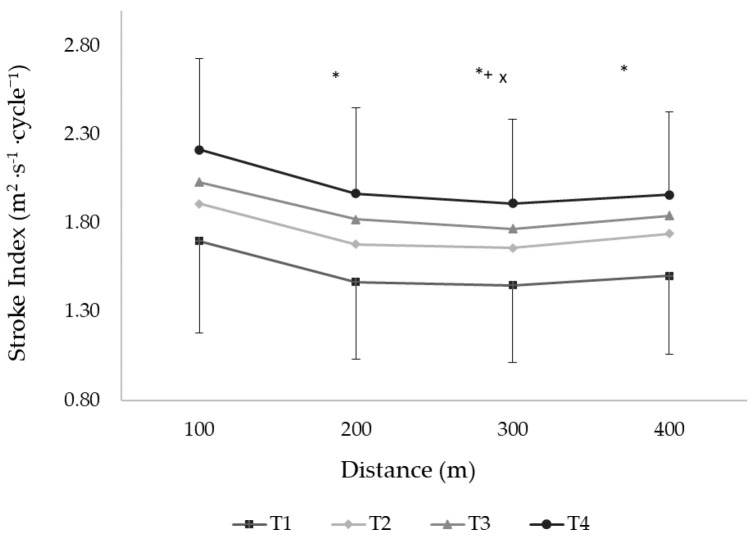
The stroke index in each 100 m split of the 400 m front crawl effort at testing moments T1, T2, T3, and T4. T1: beginning of the first macrocycle; T2, T3, and T4: end of the first, second, and third macrocycles, respectively. * and +: *p* < 0.05 compared to 100 m and 400 m. ^x^: *p* < 0.05 compared to 200 m partial.

The mean SR of the 400 m front crawl increased at T3 and T4 compared to T1 (*F*_3.96_ = 3.36, *p* = 0.02, Table 3). This variable decreased in the second, third, and fourth partials compared to the first 100 m split and increased in the last split independent of the testing moment (*F*_3.96_ = 90.6, *p* < 0.01, Figure 2). The SL augmented after T2, T3, and T4 compared to T1 and after T4 compared to T2 and T3 (*F*_3.99_ = 31.45, *p* < 0.01, Table 3). A decreased SL was observed after the second, third, and fourth partials compared to the first 100 m split (*F*_3.99_ = 20.57, *p* < 0.01). The SI improved in each testing moment compared to the previous one (T1 vs. T2, T2 vs. T3, and T3 vs. T4, *F*_3.99_ = 82.44, *p* < 0.01, Table 3) and declined in each 100 m split compared to the previous one in all testing moments (*p* < 0.01, Figure 3).

### 3.4. Relationships between Variables

All the measured variables, at each testing moment, are related (*p* < 0.05) with the 400 m front crawl speed (Table 4).

**Figure 4:**
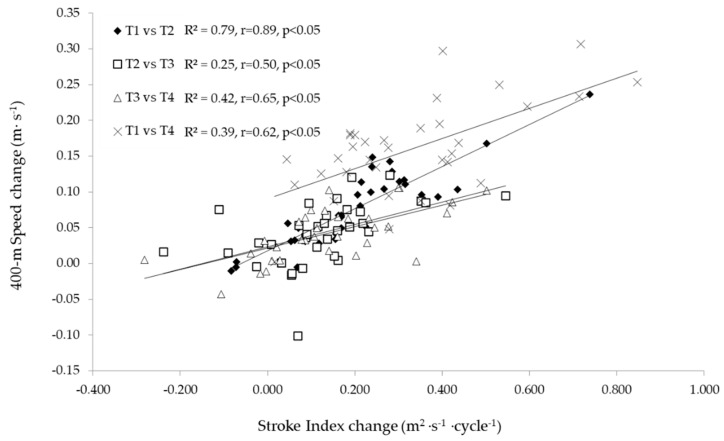
Relationship of stroke index changes and 400 m speed changes between macrocycles. T1: beginning of the general preparation period of the first macrocycle; T2, T3, and T4: end of the competitive period of the first, second, and third macrocycles.

Changes in anthropometric variables between macrocycles (such as body mass, stature, and body mass index) were not related to the 400 m speed changes (*r* = −0.05 to 0.20, *p* > 0.05). Blood lactate and blood glucose changes from T1 to T2 were related to 400 m speed changes (*r* = 0.35 and 0.36, *p* < 0.05), but subsequent changes from T2 to T3, T3 to T4, and T1 to T4 showed no relationship with 400 m speed modifications (*p* > 0.05). SR changes were not related to speed alterations (*p* > 0.05), and SL changes from T1 to T2, T3 to T4, and T1 to T4 were related to 400 m speed modifications (*r* = 60, 0.34, and 0.58, *p* < 0.05). The calculated SI is the only measured variable that is significantly related with the increase in speed between all testing moments (Figure 4).

## 4. Discussion

The purpose of the current study was to follow up on the evolution of young swimmers’ middle-distance performance (and respective physiological and biomechanical changes) along a training season. For that purpose, a 45-week longitudinal evaluation was used along the three macrocycles of the year plan and four 400 m front crawl tests (a distance commonly used in age group training, competition, and monitoring [4,10,13]) were conducted. It is known that metabolic factors provide the basis for swimming performance improvement, especially in the first macrocycle of the season [3,5,15], and that technique progression is evident along the training season [2,6,27]. However, there are very few longitudinal studies available on young swimmers, and they too lack detailed information about the interplay between the changes in performance, its determinant variables, and training contents over a full training swimming season.

We observed that performance increased by 14.6% along the three macrocycles, with a greater improvement observed in the first, followed by the second and third macrocycles. Previous studies have already reported 1.9–3.6% improvement in a 400 m test over a training season in young male and female swimmers [13,28]. In the current study, most of the anthropometric and biomechanical variables were substantially improved, especially during the first macrocycle, with ~2 and 12% stature and SI improvements observed from T1 to T2 (consisting in half of their overall improvement within the 45 weeks). Since it is well accepted that different factors contribute to young swimmers’ performance enhancement [6,16,27], the observed 400 m time improvements may also be related to training content and corresponding metabolic changes within each macrocycle. In fact, increased training intensity (through higher anaerobic loads) may alter swimming performance improvement rate [10,17,29]. As such, the ~12 and 18% rise in [La] and [Glu], despite an attenuated improvement in SI and SL during T3 compared to T2, may explain the differences in the observed performance improvement rates, in agreement with a previous study [8].

The current study relationships between the anthropometric, physiological, and biomechanical variables and the 400 m front crawl time confirmed their importance for young swimmers’ middle-distance performance (as observed before [8,10,28]). However, even if most of the measured variables correlated well with the 400 m performance, its magnitude altered between testing moments. Body mass and stature, in agreement with previous studies [13,17,28], were moderately related with performance during T1 and T2, a period where the greater improvement in these variables was observed in the current study. In addition, SL and SI displayed consistent high correlation values with middle-distance swimming, highlighting its importance for swimmers in this age group (as proposed before [5,8,15]). [La] and [Glu] variables were also well related with the 400 m speed along all the testing moments, supporting the idea that energetics and technique have a relative higher contribution to aerobic power efforts compared to body mass and stature [8,10,17]. Any differences between the magnitudes of the correlation values in the current data and the literature may be attributed to the distance selected as a performance criterion (i.e., 100, 200, vs. 400 m), the swimmer’s age, and the year training period [4,10,15].

All variables’ modifications were also examined in relation to the 400 m front crawl speed changes over the training period. SI variations were strongly related to 400 m speed increments between testing moments. This was not a surprise since it is known that the SI discriminates the swimmers’ technical ability [6,18,19], which is likely more important for longer than shorter distances [8,15,20]. The SI increased by ~23% from T1 to T4, with the greater improvement observed between T1 and T2 (as expected for the initial training macrocycle). Similar findings were reported before, recognizing SI as a factor explaining 90% of the swimming performance in distances of 100–400 m [14], which may be connected to technical improvements or increments in body dimensions [6]. A significant technique training content in the first macrocycle may have contributed to the greater improvement between T1 and T2 moments.

[La] values increased along the training season, possibly due to the anaerobic training content increment between macrocycles that led to a higher glycolytic contribution to the 400 m front crawl performances [8,22]. It should be noted that [La] variations between T1 and T2 were related to 400 m performance changes, a fact that was not observed in the second and third macrocycles. It is likely that increased training intensity led to metabolic changes within a macrocycle (12–18 weeks), which affected performance [8,29], even if the technical training contributes more than 50% to performance improvement [8]. It seems that our younger swimmers’ technique changes were more evident than those reported in adolescent swimmers [8], a fact supported by the continuous importance of SI and SL changes in all testing moments.

[Glu] values were higher at T3 and T4 compared to T2. A previous work showed that [La] and [Glu] increases are coincident [17], and another study stated that [Glu] alone may not be adequate to express training-induced improvements [30]. In the current study, [Glu] modifications were related to 400 m performance changes in a manner similar to [La] changes (presenting similar correlation values), which may indicate a metabolic connection among these metabolites. Although the current swimmers’ diet and training were controlled, a complex metabolic mechanism is connected to [Glu] regulation, making it difficult to explain the changes in this variable. In addition, post-swim [Glu] levels may be altered by the intensity and/or the duration of the swimming bouts [31]. Whatever the case, the significant [Glu] connection with performance changes between T1 and T2 indicates its importance, requiring a more in-depth evaluation in future studies.

## 5. Conclusions

The current study findings indicate that a well-designed year plan, with the training periods coinciding with the swimmers’ school calendar, leads to a significant middle-distance swimming performance improvement in young swimmers. The fact that the 400 m front crawl speed enhancement along the season is mostly connected with an increase in the values of biomechanical (SL and SI) variables suggests that when swimmers are in this age group, coaches should prioritize the swimmers’ technique development. It should be highlighted that, concomitantly with perfection in swimming skills, physiological variables (such as [Glu] and [La]) are likely important to optimize middle-distance swimming performance. Thus, coaches may combine a robust technical training in the beginning of the season with a progressive increase in training intensity across macrocycles (aiming to gradually stimulate the anaerobic metabolism) for optimal performance progression in young swimmers. In female swimmers, the phase of the menstrual cycle was not controlled, which can affect fluid retention and performance. Since this is a factor that potentially adds variability in the data, future studies should consider this issue.

## Figures and Tables

**Table 1:** Anthropometric characteristics of the young swimmers engaged in the current study.

Variables	Boys (*n* = 24)	Girls (*n* = 10)	Total Sample (*n* = 34)
Chronological age (years)	12.51 ± 0.99	11.24 ± 0.88	12.07 ± 1.14
Body mass (kg)	45.90 ± 9.54	44.26 ± 8.76	45.42 ± 9.22
Stature (m)	1.56 ± 0.11	1.52 ± 0.07	1.55 ± 0.10
Tanner stage	2.94 ± 1.04	3.05 ± 1.10	2.97 ± 1.05

**Table 2:** Swimming training content, volume, and frequency per macrocycle along a young swimmer’s training season.

Training Type	1st Macrocycle	2nd Macrocycle	3rd Macrocycle
Aerobic training (%)	91	89	85
Anaerobic training (%)	9	11	15
Technical training (%)	28	16	14
Conditional training (%)	72	84	86
Distance per week (m)	19,708 ± 4207	16,577 ± 5655	15,933 ± 5496
Training sessions per week	5.83 ± 0.56	5.80 ± 0.63	5.82 ± 0.58

**Table 3:** Changes in swimmers’ anthropometric, physiological, and biomechanical variables during the four 400 m front crawl testing moments. Mean ± SD, 95% confidence intervals (95% CI), and effect size (ES) are presented.

Variables	T1	T2	T3	T4	η^2^ (*p*)
Body mass (kg)	44.39 ± 9.33	45.42 ± 9.22 *	47.00 ± 9.21 *+	47.31 ± 9.58 *+	0.45 (0.00)
95% CI	41.14–47.65	42.51–49.20	43.79–50.21	43.96–50.65	
ES	vs. T2: 0.10	vs. T3: 0.16	vs. T4: 0.04	vs. T1: 0.30	
Height (m)	1.52 ± 0.10	1.55 ± 0.10	1.56 ± 0.10 *+	1.58 ± 0.10 *+	0.68 (0.00)
95% CI	1.49–1.56	1.51–1.59	1.53–1.60	1.54–1.61	
ES	vs. T2: 0.24	vs. T3: 0.15	vs. T4: 0.15	vs. T1: 0.54	
Body mass index (kg·m^2^)	19.05 ± 2.35	18.99 ± 2.28	19.11 ± 2.17	18.88 ± 2.28	0.03 (0.41)
95% CI	18.17–19.79	18.06–19.70	18.38–19.84	18.09–19.64	
ES	vs. T2: −0.07	vs. T3: 0.11	vs. T4: −0.10	vs. T1: −0.07	
Time 400 m (s)	432.37 ± 71.78	396.58 ± 55.00 *	381.67 ± 51.9 *+	366.66 ± 47.7 *+x	0.98 (0.00)
95% CI	407.33–457.42	377.39–415.76	363.57–399.78	350.02–383.30	
ES	vs. T2: −0.56	vs. T3: −0.28	vs. T4: −0.30	vs. T1: −1.10	
Heart rate (bpm)	154.28 ± 23.92	155.97 ± 26.62	155.67 ± 26.08	156.00 ± 26.67	0.03 (0.36)
95% CI	175.52–186.61	181.17–190.36	182.10–189.09	180.04–189.96	
ES	vs. T2: 0.23	vs. T3: −0.01	vs. T4: −0.05	vs. T1: 0.16	
Blood lactate (mmol·L^−1^)	6.04 ± 2.33	6.32 ± 2.51	7.16 ± 2.67 *	7.94 ± 2.74 *+	0.21 (0.00)
95% CI	5.22–6.85	5.44–7.19	6.23–8.10	6.98–8.89	
ES	vs. T2: 0.12	vs. T3: 0.33	vs. T4: 0.28	vs. T1: 0.75	
Blood glucose (mmol·L^−1^)	110.30 ± 15.88	102.29 ± 19.68	122.88 ± 18.35 *+	118.03 ± 27.31 +	0.26 (0.00)
95% CI	104.75–115.84	95.43–109.16	116.48–129.29	108.50–127.56	
ES	vs. T2: −0.45	vs. T3: 1.08	vs. T4: −0.21	vs. T1: 0.36	
Rating of perceived exertion	14.91 ± 1.93	14.88 ± 2.40	15.09 ± 2.19	14.59 ± 2.44	0.01 (0.68)
95% CI	14.24–15.58	14.05–15.72	14.32–15.85	13.74–15.44	
ES	vs. T2: −0.01	vs. T3: 0.09	vs. T4: −0.22	vs. T1: −0.15	
Stroke rate (cycles·min^−1^)	36.47 ± 3.83	37.14 ± 4.96	37.75 ± 5.19 *	37.63 ± 4.87 *	0.09 (0.02)
95% CI	35.01–37.69	35.61–38.66	36.00–39.14	36.28–38.97	
ES	vs. T2: 0.20	vs. T3: 0.07	vs. T4: 0.02	vs. T1: 0.31	
Stroke length (m·cycle^−1^)	1.58 ± 0.24	1.67 ± 0.20 *	1.72 ± 0.24 *	1.78 ± 0.22 *+x	0.48 (0.00)
95% CI	1.49–1.66	1.60–1.74	1.63–1.80	1.70–1.85	
ES	vs. T2: 0.41	vs. T3: 0.22	vs. T4: 0.26	vs. T1: 0.87	
Stroke index (m^2^·s^−1^·cycle^−1^)	1.53 ± 0.46	1.73 ± 0.42 *	1.86 ± 0.48 *+	1.99 ± 0.47 *+x	0.71 (0.00)
95% CI	1.37–1.69	1.59–1.88	1.69–2.02	1.83–2.16	
ES	vs. T2: 0.47	vs. T3: 0.28	vs. T4: 0.29	vs. T1: 1.00	

T1: beginning of the general preparation period of the first macrocycle; T2, T3, and T4: end of the competitive period of the first, second, and third macrocycles, respectively. *, +, and x: *p* < 0.05 compared to T1, T2, and T3.

**Table 4:** Correlation values between anthropometric, physiological, and biomechanical variables and the 400 m front crawl performance at the four testing moments of the season.

Variables	T1	T2	T3	T4
Body mass (kg)	0.35 *	0.35 *	0.26	0.34
Stature (m)	0.39 *	0.40 *	0.33	0.41 *
Blood lactate (mmol·L^−1^)	0.50 *	0.72 *	0.62 *	0.55 *
Blood glucose (mmol·L^−1^)	0.41 *	0.55 *	0.54 *	0.50 *
Stroke rate (cycle·min^−1^)	0.42 *	0.63 *	0.45 *	0.52 *
Stroke length (m·cycle^−1^)	0.77 *	0.59 *	0.63 *	0.67 *
Stroke index (m^2^·s^−1^·cycle^−1^)	0.93 *	0.91 *	0.91 *	0.92 *

T1: beginning of the general preparation period of the first macrocycle; T2, T3, and T4: end of the competitive period of the first, second, and third macrocycles. *: *p* < 0.05.

## Data Availability

Data presented in this study are available on request from the corresponding author. The data are not publicly available due to ethical reasons.
